# Sorafenib plus memory-like natural killer cell immunochemotherapy boosts treatment response in liver cancer

**DOI:** 10.1186/s12885-024-12718-4

**Published:** 2024-09-30

**Authors:** Aydin Eresen, Zigeng Zhang, Guangbo Yu, Qiaoming Hou, Zhilin Chen, Zeyang Yu, Vahid Yaghmai, Zhuoli Zhang

**Affiliations:** 1 Department of Radiological Sciences, University of California Irvine, Irvine, CA USA; 2 Department of Biomedical Engineering, University of California Irvine, Irvine, CA USA; 3 Department of Biological Sciences, University of Southern California, Los Angeles, CA USA; 4 Information School, University of Washington, Seattle, WA USA; 5 Chao Family Comprehensive Cancer Center, University of California Irvine, Irvine, CA USA; 6 Department of Pathology and Laboratory Medicine, University of California Irvine, Irvine, CA USA

**Keywords:** Combination therapy, Liver cancer, Memory-like natural killer cell immunotherapy, Magnetic resonance imaging, Sorafenib

## Abstract

**Background:**

Heterogeneity of hepatocellular carcinoma (HCC) presents significant challenges for therapeutic strategies and necessitates combinatorial treatment approaches to counteract suppressive behavior of tumor microenvironment and achieve improved outcomes. Here, we employed cytokines to induce memory-like behavior in natural killer (NK) cells, thereby enhancing their cytotoxicity against HCC. Additionally, we evaluated the potential benefits of combining sorafenib with this newly developed memory-like NK cell (*p*NK) immunochemotherapy in a preclinical model.

**Methods:**

HCC tumors were grown in SD rats using subcapsular implantation. Interleukin 12/18 cytokines were supplemented to NK cells to enhance cytotoxicity through memory activation. Tumors were diagnosed using MRI, and animals were randomly assigned to control, *p*NK immunotherapy, sorafenib chemotherapy, or combination therapy groups. NK cells were delivered locally via the gastrointestinal tract, while sorafenib was administered systemically. Therapeutic responses were monitored with weekly multi-parametric MRI scans over three weeks. Afterward, tumor tissues were harvested for histopathological analysis. Structural and functional changes in tumors were evaluated by analyzing MRI and histopathology data using ANOVA and pairwise T-test analyses.

**Results:**

The tumors were allowed to grow for six days post-cell implantation before treatment commenced. At baseline, tumor diameter averaged 5.27 mm without significant difference between groups (*p* = 0.16). Both sorafenib and combination therapy imposed greater burden on tumor dimensions compared to immunotherapy alone in the first week. By the second week of treatment, combination therapy had markedly expanded its therapeutic efficacy, resulting in the most significant tumor regression observed (6.05 ± 1.99 vs. 13.99 ± 8.01 mm). Histological analysis demonstrated significantly improved cell destruction in the tumor microenvironment associated with combination treatment (63.79%). Interestingly, we observed fewer viable tumor regions in the sorafenib group (38.9%) compared to the immunotherapy group (45.6%). Notably, there was a significantly higher presence of NK cells in the tumor microenvironment with combination therapy (34.79%) compared to other groups (ranging from 2.21 to 26.50%). Although the tumor sizes in the monotherapy groups were similar, histological analysis revealed a stronger response in *p*NK cell immunotherapy group compared to the sorafenib group.

**Conclusions:**

Experimental results indicated that combination therapy significantly enhanced treatment response, resulting in substantial tumor growth reduction in alignment with histological analysis.

## Background

One of the most challenging cancers globally, liver cancer is expected to surpass 1 million diagnoses annually [1]. Hepatocellular carcinoma (HCC), accounting for up to 90% of liver cancer cases, is the seventh most prevalent cancer worldwide, and the second leading cause of cancer-related deaths [2]. The treatment of HCC has significantly evolved with the introduction of curative-intent options such as liver transplantation, surgical resection, and local ablative therapies, which collectively offer a promising 5-year survival rate of approximately 70% [3]. Minimally invasive surgical techniques have revolutionized surgical resection while improvements in intraoperative and perioperative management strategies have expanded patient eligibility for surgical intervention; however, clinical outcomes remain comparable to traditional surgical techniques [4]. Despite ongoing efforts, only 40% of patients with early-stage tumors are eligible for potentially curative approaches. Most HCC cases are diagnosed at advanced stages, where the median survival rate is less than one year.

Emerging as a viable curative option for unresectable HCC patients, local ablative therapies offer promising outcomes leading to 46% of 3-year recurrence-free survival and 76% of overall survival (OS) rates for HCC tumors 3 cm or smaller [5]; however, therapeutic responses for the ablative therapies against larger tumors are significantly reduced [6]. For patients with unresectable HCC lacking vascular invasion, extrahepatic spread, or significant liver dysfunction transarterial chemoembolization (TACE) remains the recommended first-line therapy that objective responses can exceed 50% and improves the overall survival significantly [7, 8]. For TACE unsuitable patients, recently approved systemic therapies offer a promising alternative treatment strategy. Clinical trials have reported objective responses in approximately 30% of patients treated with atezolizumab plus bevacizumab, 20% with durvalumab plus tremelimumab, and 20% with lenvatinib monotherapy [9–11]. Recent achievements in HCC treatment are marked by the IMbrave150 study, which established the combination of atezolizumab and bevacizumab as the first-line standard of care for unresectable HCC. The study demonstrated superior OS, progression-free survival (PFS), and objective response rates when compared to sorafenib. These developments underscore both the progress made and the lingering debates within the field, emphasizing the necessity for ongoing research to optimize and personalize treatment strategies for HCC [10, 12]. Despite significant advancements, analysis suggests that adjuvant atezolizumab and bevacizumab may offer the most benefit to a specific subgroup of patients, rather than the entire HCC population [13]. This highlights the critical need for improved patient stratification to better align clinical outcomes with specific molecular characteristics of the tumor.

For two decades, sorafenib has served as the standard care for patients with advanced-stage HCC [13], establishing itself as one of the most effective single-drug therapies available [1]. As the first tyrosine kinase inhibitor receiving FDA approval for systemic treatment of HCC, sorafenib inhibits proliferation and angiogenesis by suppressing the activity of serine-threonine kinases Raf-1 and B-Raf, and receptor tyrosine kinase activity of VEGFR-1, 2, and 3 and PDGFR-β [14–16]. Sorafenib serves as the standard treatment for advanced-stage HCC. However, its effectiveness is limited by the complex and diverse nature of the tumor, as well as the activation of multiple signaling pathways that promote cancer cell survival and growth [17, 18]. This limited patient response underscores the urgent need for developing novel combination strategies to suppress tumor progression and improve survival rates.

The remarkable success of immunotherapy in the treatment of various other cancers has led to investigations for HCC treatment [19]. During the last decade, the FDA has approved several immune checkpoint inhibitors for the treatment of cancers including HCC [11, 12]. The recent FDA approval of the NK-92 cell line for clinical use improved the attention of the natural killer (NK) cell immunotherapy strategies. Over the decade, NK cell immunotherapy, as an innate immune system, has shown promise in becoming a potent and well-tolerated therapy in managing a wide range of malignancies [20]. Previous research has shown that NK cells have immunological memory, allowing them to recall earlier exposure to specific memory stimuli such as antigens, cytomegalovirus, or cytokines [21]. In-vitro supplementation of IL-12/15 and IL-18 cytokines has been investigated in mice and human cell lines to enhance cytotoxicity for hematological malignancies [22, 23]. Mahgoub et al. expressed increasing cytotoxic function of NK cells following supplementation with IL-2 and IL-15 cytokines against HCC cells in combination with cetuximab [24]. This suggests that *p*NK cells hold promise as a strategy to improve the persistence and effectiveness of NK cell therapy in vivo.

Resistance to chemotherapeutic drugs presents a significant challenge for HCC treatment, frequently diminishing the efficacy of standard therapies [25]. The concurrent use of NK cell immunotherapy with sorafenib holds promise in overcoming such resistance by enhancing the immune response against cancer cells [26]. The combination harnesses capability of immune system to recognize and destroy cancer cells, potentially enhancing treatment efficacy and patient outcomes. The potential of combining sorafenib and NK cell immunotherapy for HCC is hindered by conflicting reports of both synergistic and inhibitory effects [27–32]. While earlier studies suggested the potential of sorafenib to enhance NK cell function [33–35], these findings lack consensus. A recent study demonstrated that combining sorafenib with *p*NK significantly enhanced their cytotoxicity against HCC cells compared to other treatment combinations, including single therapies [36]. This suggests that *p*NK cells hold promise as a strategy to improve the persistence and effectiveness of NK cell therapy in vivo. In this study, we further investigated the sorafenib plus transcatheter intraarterial administration of *p*NK in a rat HCC model and evaluated early therapeutic response using anatomical MRI and histopathological analysis.

## Methods

### Cell culture and tumor model development

The N1-S1 cell line is widely utilized in the development of HCC research for studying critical aspects for elucidating HCC progression and developing efficacious therapeutic strategies due to its distinctive attributes that effectively bridge the gap between in vitro and in vivo investigations [37]. The syngeneic nature of the N1-S1 line with Sprague Dawley rats obviates graft rejection, enabling the implantation of HCC cells and subsequent study of tumor development within an immunocompetent host that constitutes a significant advantage over xenograft models that rely on immunodeficient mice. Therefore, the unique confluence of syngeneic origin, rapid tumor formation, and well-characterized properties elevates the N1-S1 cell line to a pivotal role in vitro HCC tumor modeling. For our study, the N1-S1 rat HCC cell line was purchased from ATCC (Manassas, VA), and the rat NK cell line (RNK-16) was kindly provided by Thomas L. Olson (University of Virginia, Charlottesville, VA). Both cell lines were cultured according to the suggested protocols and incubated at 37 °C in a humid atmosphere containing 5% CO_2_ and 95% air. Tumor cell viability (> 90%) was validated before tumor cell implantation. For generation of *p*NK cells, RNK-16 cells were cultured in fresh medium supplemented with 5 ng/mL of IL-12 and 40 ng/mL of IL-18 cytokines for 24 h [36], rinsed with PBS, and allowed to rest for another 24 h before experiments. Cell viability was measured using Countess II (Life Technologies, Carlsbad CA).

All procedures were conducted in adherence to the animal protocol authorized by the Institutional Animal Care and Use Committee of our institution. The liver of the subjects was exposed via incision and 1.5 × 10^6^ N1-S1 cells were injected subcapsular under anesthesia induced via 2% isoflurane with 3 L/min of oxygen. Hemostatic gauze was placed with medium pressure to prevent leakage and the incision was closed by performing a two-layer closure technique. Pain medicine (0.05 mg/kg of buprenorphine, and 2 mg/kg of meloxicam) was administered subcutaneously, and animals were allowed to recover in cages with food and water available ad libitum. Tumors were allowed to grow while the animals were observed daily for any signs of distress.

### Therapeutic strategy

Upon tumors reaching an approximate size of 5 mm on T1w and T2w MRI, twenty-four animals were randomly assigned into the control group (*n* = 6), sorafenib group (*n* = 6), *p*NK cell immunotherapy group (*n* = 6), and sorafenib plus *p*NK (combination) immunochemotherapy group (*n* = 6). For the sorafenib treatment group, a stock solution of 20 mg/ml was prepared by diluting sorafenib tosylate in a 1:1 solution of castor oil (Kolliphor^®^ EL, Sigma Aldrich, St Louis, MO) and 95% ethanol. The dosage of the sorafenib was determined according to previous studies [38–40]. A daily dosage of 10 mg/kg sorafenib was administered using a bulb-tipped gastric gavage needle while animals were restrained. Afterward, the rats were placed in their cages and observed for 10 min to identify any indications of difficulty breathing or discomfort. The animals in *p*NK cell immunotherapy and combination groups underwent catheterization of the proper hepatic artery following the procedure described by Sheu et al. [41], three days after the baseline scans. Briefly, the portal triad above the first loop of the duodenum was surgically exposed, the common hepatic artery was temporarily ligated with a 2 − 0 suture to prevent bleeding, and a 4 − 0 suture was utilized to permanently ligate the gastroduodenal artery to prevent backward flow of the cells to the bowels. A 24G microcatheter (Terumo SurFlash^®^, Somerset, NJ) was inserted distal to this ligation point in the gastroduodenal artery and then guided into the proper hepatic artery. Subsequently, 0.1 mL of heparin was infused through a catheter, followed by the administration of 10^7^*p*NK cells with 0.5 mL of PBS and 0.2 mL of saline flush [42]. The catheter was removed, and a 4 − 0 suture was employed to permanently litigate the gastroduodenal artery.

### MRI and histology analysis

The animals were imaged via 3T Philips MRI with a commercial wrist coil under anesthesia. The animals underwent weekly MRI examinations until 2 weeks posttreatment or recruitment to monitor tumor growth and evaluate treatment response in vivo. The HCC tumors were located with a T1w MRI sequence (Repetition time (TR): 200 ms, echo time (TE): 2.45 ms, slice thickness (ST): 2 mm (no gap), flip angle (FA): 90º, field of view (FOV): 50 × 50 mm^2^, number of signal acquisitions (NSA): 4). MRI sequences and parameters were as follows: (a) T2w: TR: 3500 ms, TE: 63.177, ST: 2 mm (no gap), NSA: 4, FOV: 50 × 50 mm^2^, NSA: 4; (b) T1w: TR: 200 ms, TE: 2.45 ms, ST: 2 mm (no gap), FA: 90º, FOV: 50 × 50 mm^2^, NSA: 4. ITK-SNAP (v.4.0) was used for outlining the tumor tissues based on T1w and T2w MRI data and translated to all the acquired images following affine transformation [43]. Tumor growth was measured as follows, $\:{\varDelta\:D}_{i,j}=\left({D}_{i}-{D}_{j}\right)/{D}_{j}$ where $\:{D}_{i}$ and $\:{D}_{j}$ are the tumor dimensions in the longest axis at *i*^th^ and *j*^th^ time points. MATLAB (v.9.10, MathWorks, Natick, MA) was employed for image processing and quantitative analysis.

When the animals reached the study endpoint, livers were harvested, and a 4 mm thick tissue block was immediately fixed in 10% formalin. Tissues were embedded in paraffin wax and 5 *µ*m sections of paraffin-embedded liver tissues were stained with H&E (cell viability) and CD56^+^ antibody (NK cells). The histology slides were digitized via the Hamamatsu whole slide scanner and analyzed with QuPath (v0.4.3) [44]. The blinded researchers analyzed five randomly selected regions to quantify the viable tumor cells and NK cells in the corresponding images at 40× magnification.

### Statistical analysis

The statistical analyses were conducted using GraphPad Prism (La Jolla, CA). One-way ANOVA and pairwise T-tests were performed to evaluate the significance (*p* < 0.05) of the findings. Data are expressed as the mean ± standard deviation or standard error. The data generated in this study are available upon request from the corresponding author.

The characteristics of the tumor microenvironment were captured using T1w and T2w MRI data, from which first-order statistical features (mean, standard deviation, third moment, entropy, kurtosis, and skewness) were extracted. The correlation between MRI data and histological measurements was assessed through multivariable analysis of the intensity distribution characteristics of the T1w and T2w MRI data. We explored the predictive potential of these features in forecasting histological tumor markers obtained via pathological analysis. Specifically, we investigated the association between quantitative structural MRI characteristics and histopathological markers for the noninvasive assessment of the tumor microenvironment.

## Results

### In vitro cytotoxicity analysis

**Fig. 1:**
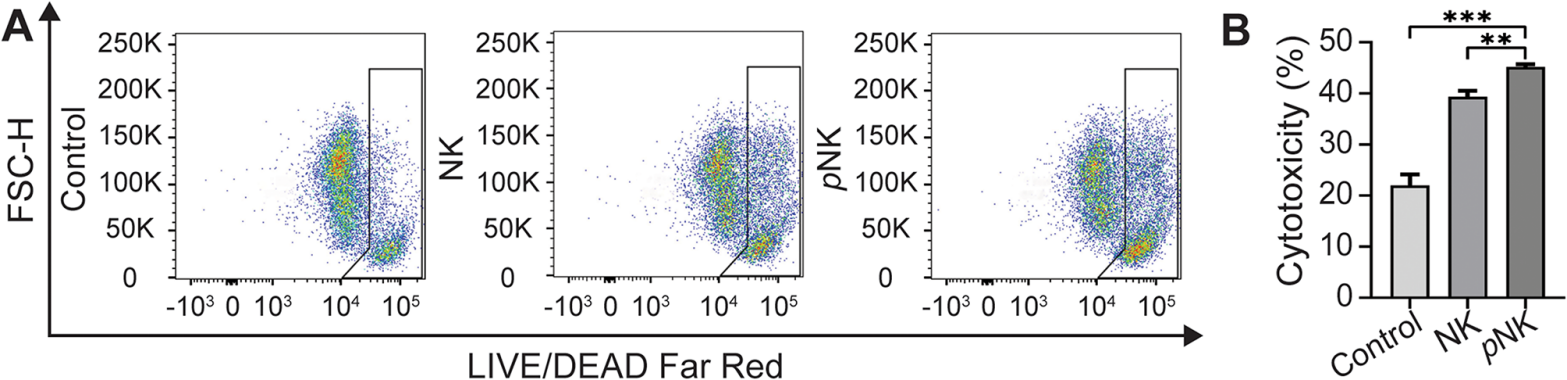
Development of memory-like rat NK cells (pNK) via supplementation of IL-12 plus IL-18 through optimization of NK cell viability and cytotoxicity. The concentration of IL-12 (**A**) cytokines was associated with more potent cytotoxicity than IL-18 (**B**) as well as weakening cell viability with more IL-12 (**C**). (**: *p* < 0.01, ***: *p* < 0.001)

The efficacy of *p*NK cells was evaluated by assessing cytotoxicity against the N1-S1 rat HCC cell line, using a 10:1 effector-to-target ratio. Following a 24-hour cytokine supplementation of NK cells, tumor cell viability was analyzed across the control, NK, and *p*NK groups using flow cytometry. The results demonstrated a significant increase in cell death efficacy in the treatment groups (NK and *p*NK) compared to the control group (*p* < 0.001, Fig. 1). Furthermore, cytokine treatment significantly enhanced the cytotoxic function of NK cells against HCC cells (*p* < 0.01). The observed percentages of cell death were 21.98 ± 2.16% in the control group, 39.34 ± 0.17% in the NK group, and 45.18 ± 0.57% in the *p*NK group. These findings collectively suggest that *p*NK cells can significantly enhance cytotoxic function while maintaining effector cell viability.

### In vivo tumor size measurement on MR images

**Fig. 2:**
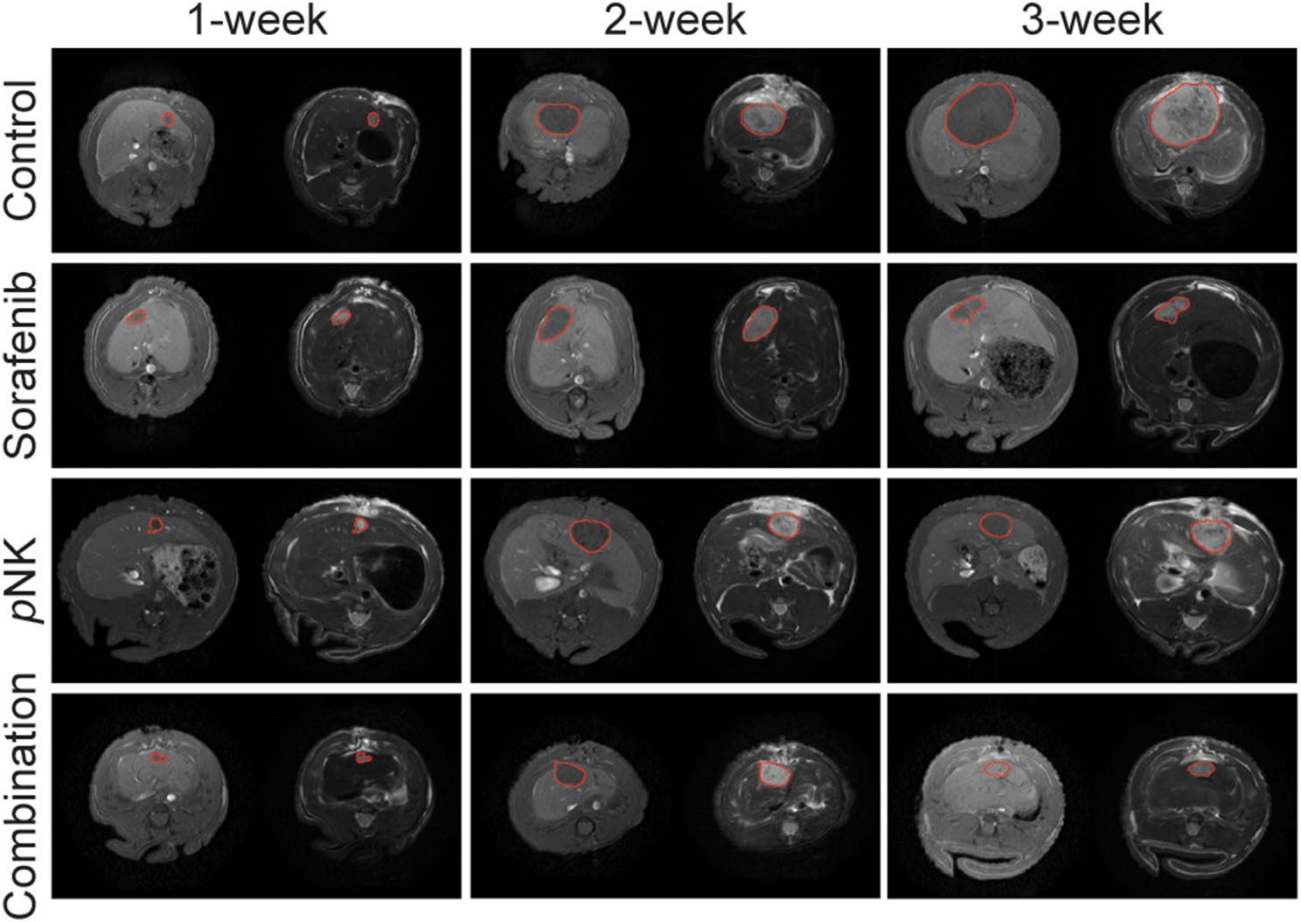
Representative MRI data to demonstrate the tumor growth pattern in different groups. Tumors were outlined (red circle) at each time point (baseline; post 1-week and post 2-week treatment) based on T1w and T2w MRI data. In each pair, T1w (left) and T2w (right) MRI were utilized to determine tumor size and quantitative changes

The morphology of the HCC tumors and therapeutic outcomes following different treatment strategies were investigated by performing a preclinical study focusing on an orthotopic rat tumor model. Animals were anesthetized and liver regions were exposed via abdominal incision. Approximately 1.5 million N1-S1 cells injected into the subcapsular regions of the liver were grown for five days and subjects were assigned to one of the four groups (control, *p*NK cell immunotherapy, sorafenib treatment, and combination therapy) following validation of the tumor size via baseline MRI. In our experiments, all animals implanted with tumor cells developed tumors after 5 days and were detected by multi-parametric MRI acquisitions. The tumors were quantitatively evaluated based on T1w and T2w MRI characteristics at each time point and quantitatively assessed for longitudinal changes. Tumor dimensions were measured by an expert radiologist following weekly MRI scans. The representative weekly T1w and T2w MRI data of HCC tumors from the control, sorafenib therapy, *p*NK cell immunotherapy, and combination groups are presented in Fig. 2 to qualitatively evaluate the disease characteristics. HCC tumor sizes at baseline were similar in all the groups (5.27 ± 1.74 mm), without any significant difference among the groups (*p* = 0.16). After diagnosis of tumors, subjects were randomly assigned to groups and started to receive appropriate therapeutic regimens.

**Fig. 3:**
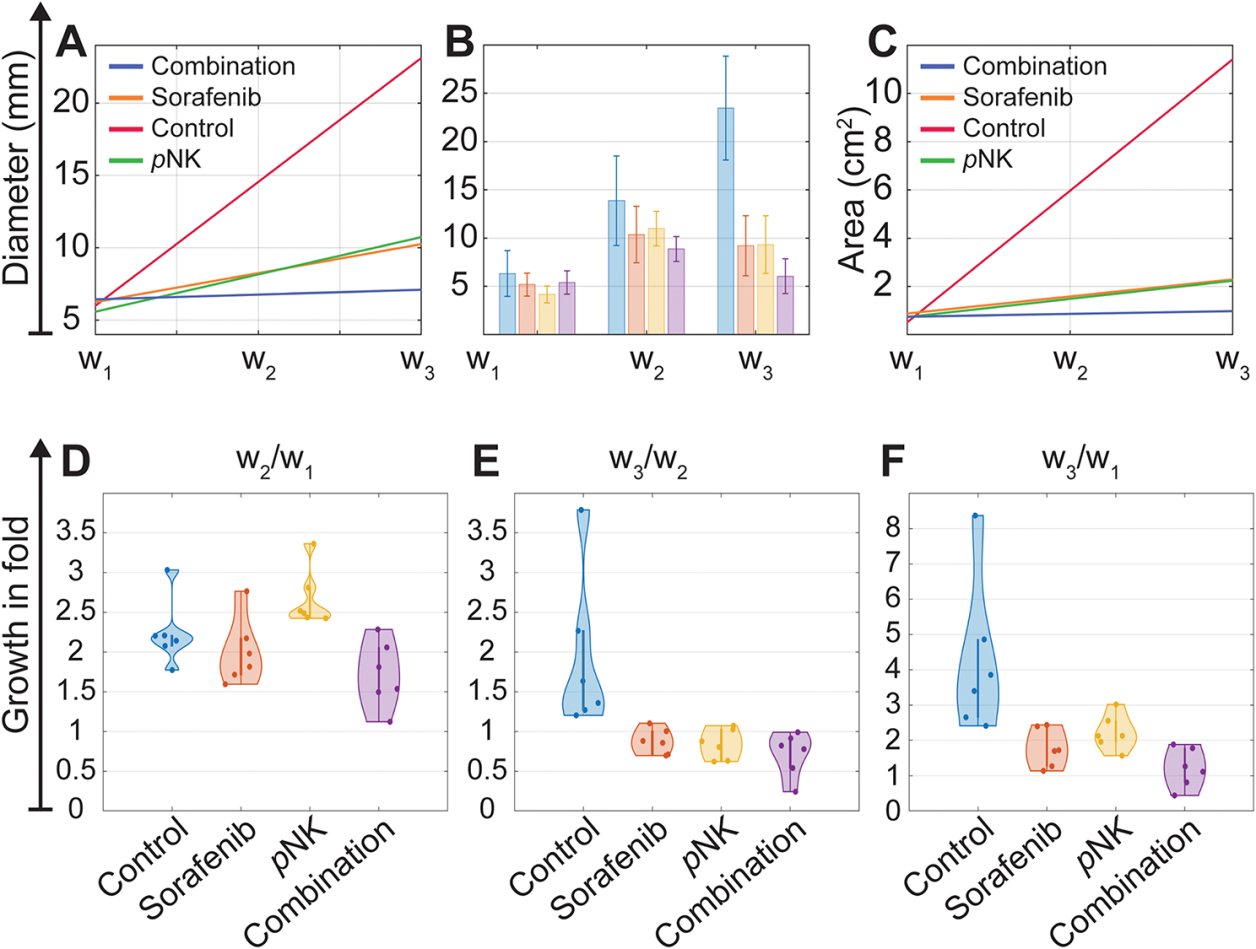
Comparison of tumor growth patterns associated with different treatment strategies. The tumors were expanded aggressively compared to treatment groups in which combination therapy demonstrated the most effective therapeutic response during the experimental timeline (**A**-**C**). The standard deviation of the tumor diameter is visualized in **B.** The disparities in tumor growth rates continued to widen, with tumors subjected to combination therapy consistently maintaining their smaller size (**D**-**F**)

The animals in the sorafenib and combination groups received sorafenib drug solution orally each day after the baseline scan for seven days. Moreover, subjects in the control, pNK cell immunotherapy, and combination groups received either saline or *p*NK cell solution according to their assignment four days after the baseline. In the first week of treatment (Fig. 3), no significant tumor area change was observed between the treatment and control groups. However, the combination group facilitated the most effective therapeutic response among all treatment strategies (*p* = 0.24 vs. *p*= [0.44–0.48]). The tumor dimension reached 8.86 ± 1.42 mm at its longest point, whereas larger tumors were observed in the control (13.88 ± 5.08 mm), sorafenib (10.36 ± 3.20 mm), and *p*NK groups (10.98 ± 1.94 mm). Subjects in the sorafenib group exhibited better responses compared to those in the *p*NK cell immunotherapy group (*p* = 0.014), potentially due to slower structural changes in tumors initiated by the immune response. Moreover, tumor area evaluation also highlighted the stronger tumor burden in the *p*NK cell immunotherapy group compared to the sorafenib group. In the second week of treatment, the changes in tumor structure were readily apparent. The subjects in the combination group had smaller tumors (8.86 ± 1.42 mm) than those in the control (23.45 ± 5.9 mm), sorafenib (10.36 ± 3.20 mm), and *p*NK cell immunotherapy (10.98 ± 1.94 mm) groups, while the growth rate gap between the combination and other groups continued to expand substantially (*p* < 0.05). The average tumor area receiving combination therapy demonstrated significant regression, reaching substantial levels of reduction (38.73%), whereas tumor burden in the monotherapy groups showed a slower response to treatment (sorafenib: 24% reduction, *p*NK: 16% reduction). Conversely, untreated HCC tumors continued to grow, albeit at a decelerating rate, with a marked increase in size observed (278.70%). There was no significant difference between the sorafenib and *p*NK cell immunotherapy groups (*p* = 0.1784), suggesting a viable immunotherapy response; however, sorafenib demonstrated a significantly improved response compared to the control group (*p* = 0.0394). These results indicate that sorafenib plus *p*NK cell immunochemotherapy (combination therapy) could stall tumor growth.

### Histopathological analysis

**Fig. 4:**
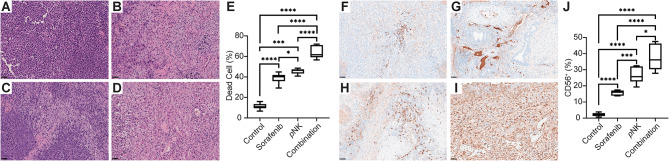
Histology analysis. The tumors treated with combination therapy demonstrated significantly fewer viable cells (**A**-**E**) and higher NK cell migration in the tumor microenvironment than the monotherapy and control groups (**F**-**J**). Although the tumor size was similar, the destruction in the tumor microenvironment was stronger among those who received *p*NK cell immunotherapy than sorafenib treatment. (*: *p* < 0.05, **: *p* < 0.01, ***: *p* < 0.001, ****: *p* < 0.0001)

The viability of the tumor cells was measured by counting the viable cells on five randomly selected different regions of the H&E-stained digital histology slides (Fig. 4A-D). One-way ANOVA demonstrated a significant difference among the groups (*p* < 0.0001), and pairwise T-tests indicated that HCC treated with sorafenib plus *p*NK cell immunotherapy facilitated robust cell death compared to all other groups (*p* < 0.0001). HCC in the *p*NK group exhibited a stronger therapeutic response than that in the sorafenib group (*p* = 0.027), while both groups showed significant advancement in cell death (*p* < 0.001). The pairwise comparison analysis of tumor viability with different treatment strategies is presented in Fig. 4E. NK cell migration to HCC tumors was measured by counting CD56^+^ cells (Fig. 4F-I). One-way ANOVA indicated a significant change in NK cell migration with sorafenib or *p*NK cell immunotherapy (*p* < 0.001). A significantly increased NK cell presence was observed in the combination group compared to the other monotherapy and control groups (*p* < 0.02). Moreover, HCC treated with *p*NK cell immunotherapy had stronger NK cell migration than sorafenib treatment (*p* = 0.027) in which both groups demonstrated significantly increased NK cells within tumors compared to untreated tumors (*p* < 0.003). Figure 4J visualizes the pairwise comparison of the CD56^+^ cells in all groups. The results supported advanced therapeutic outcomes via a combination of sorafenib and *p*NK cell immunochemotherapy in HCC subjects.

### Correlation of MRI and histopathological marker

In our analysis, we observed an increase in entropy values among the treatment groups, with sorafenib and combination treatments leading to significant changes that reflect the more complex tumor microenvironment associated with therapeutic effects in tumor regions (1.513 vs. [2.232–2.538]), with the sorafenib and combination group exhibiting strong entropy. These increased entropy values indicate a higher degree of randomness and heterogeneity within the tumor, suggesting that the treatments effectively disrupt the uniformity of tumor cell populations. Moreover, the treated tumors exhibited a more symmetrical intensity distribution, indicating a global treatment response. Combination therapy showed the lowest skewness among the groups (1.003 ± 0.277, *p* < 0.05), followed by the sorafenib and NK cell immunotherapy groups which can be interpreted as a more uniform treatment effect across the tumor mass. This uniformity may be indicative of widespread tumor cell death or alterations in tumor architecture. Untreated tumors demonstrated significantly higher kurtosis, which is potentially associated with increased tumor cell density in the tumor microenvironment in which elevated kurtosis reflects the presence of extreme intensity values, indicating dense cellular regions or areas with significant pathological features. In contrast, treated tumors exhibited higher signal magnitudes and larger standard deviations, indicative of a more complex tumor microenvironment. These variations in signal magnitude and standard deviation suggest the presence of diverse tissue types and responses within the treated tumors, pointing to a heterogeneous treatment effect. Furthermore, we observed a highly asymmetrical distribution of MRI data in the combination therapy group, followed by the sorafenib and NK cell immunotherapy groups, highlighting the heterogeneity of the tumor microenvironment following treatments. The asymmetry in the intensity distribution, characterized by skewness and kurtosis, underscores the differential impact of various therapies on the tumor microenvironment. Combination therapy may induce a more varied response, affecting different tumor regions in distinct ways.

**Fig. 5:**
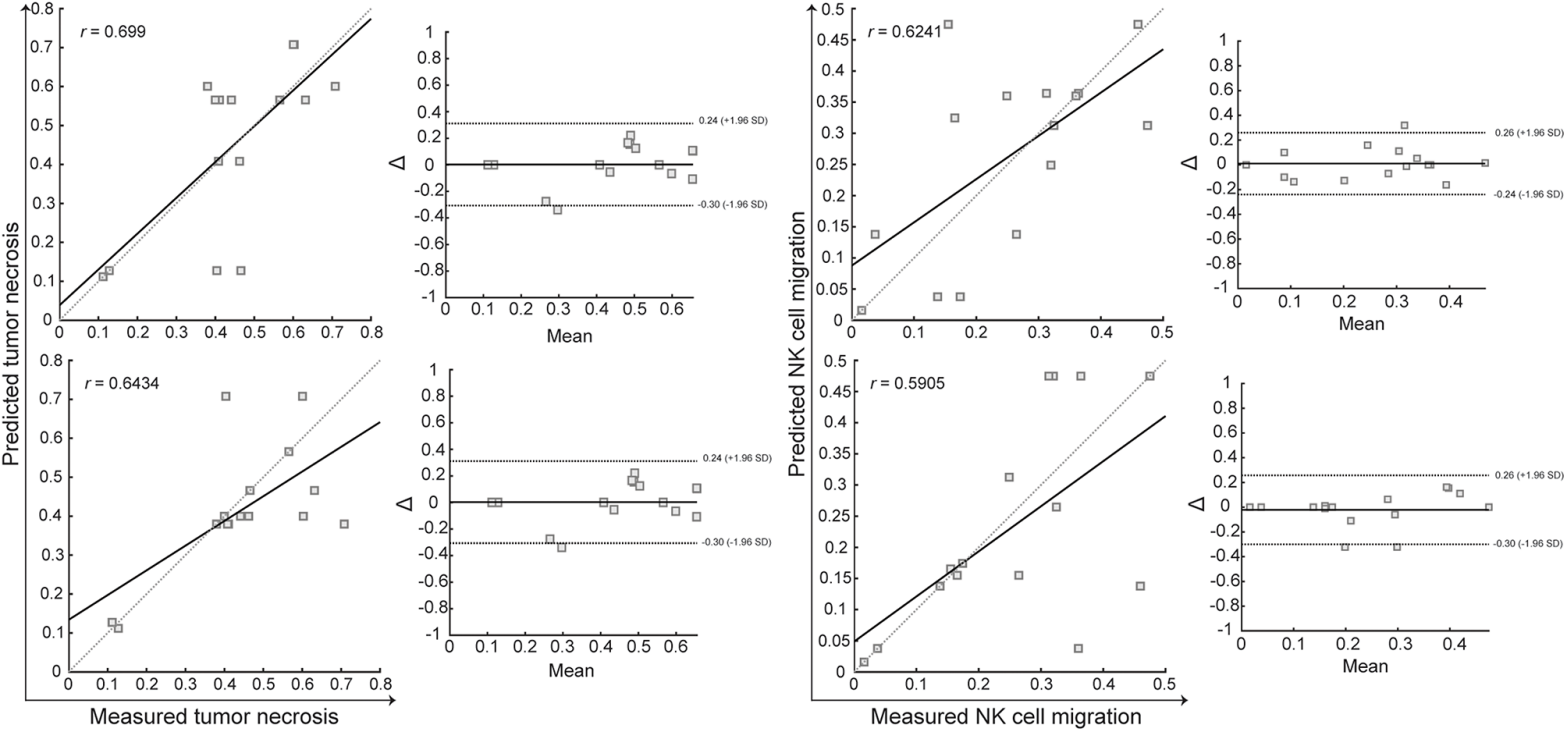
The prediction of tumor necrosis and NK cell migration within the tumor microenvironment, as measured by histological tumor markers, was assessed using histogram-based measurements of T1w (**A** and **E**) and T2w (**B** and **F**) MRI data. Bland-Altman plots were utilized to evaluate the agreement between the histological gold-standard measurements and the MRI-derived predictions of tumor necrosis and NK cell migration from T1w (**C** and **G**) and T2w (**D** and **H**) data. While T1w MRI measurements correlated well with both tumor necrosis and NK cell migration, T2w MRI-derived models exhibited weaker predictive power

To gain deeper insights into the effectiveness of therapies, we further investigated the complex interaction between tumor measurements and histopathological evaluations of the tumor microenvironment. The final logistic regression model for T1w MRI, utilizing three variables (mean, entropy, and skewness), demonstrated a strong positive correlation with the percentage of tumor necrosis (*r* = 0.699) and a mean squared error (MSE) of 0.3479 (Fig. 5A and C). In comparison, a regression model incorporating four histogram measurements (mean, standard deviation, third moment, and kurtosis) exhibited a slightly lower correlation with necrotic tissue measurements from histology (*r* = 0.6434) and a higher MSE of 1.0223 (Fig. 5B and D).

For assessing NK cell migration, more complex regression models were generated. A model including four predictors from T1w MRI data (mean, third moment, entropy, and kurtosis) resulted in a positive correlation (*r* = 0.6241) with an MSE of 1.0774 (Fig. 5E and G). In contrast, a model with five predictors extracted from T2w MRI data (standard deviation, third moment, entropy, kurtosis, and skewness) showed a weaker correlation (*r* = 0.5905) and a higher error rate (MSE = 1.3903) (Fig. 5F and H).

## Discussion

In this current study, we investigated the potential benefit of the combination of sorafenib and *p*NK cell immunochemotherapy against HCC. Our findings revealed significant structural and morphological improvements within the tumor microenvironment following combination treatment. Furthermore, the combination therapy appeared to impede tumor progression and facilitate enhanced NK cell infiltration, suggesting a heightened anti-tumor immune response. To augment the cytotoxic activity of *p*NK cells, we employed IL-12/18 cytokine stimulation due to their well-documented capacity to activate the immune system against tumors. While the clinical translation of IL-12/18 for HCC therapy remains an active area of investigation, advancements in delivery methods and combination strategies hold promise for their future application for HCC treatment.

The therapeutic landscape for first-line treatment of advanced HCC has undergone a remarkable transformation in recent years [45]. While the Barcelona Clinic Liver Cancer (BCLC) staging system remains the cornerstone for guiding therapy selection, meticulously tailoring interventions to disease severity, the emergence of immune checkpoint inhibitors (ICIs) has ushered in a paradigm shift in treatment efficacy. Atezolizumab, tremelimumab, durvalumab, and tislelizumab offer patients with advanced HCC demonstrably improved OS and PFS compared to traditional therapies like sorafenib [46–48]. Furthermore, researchers are actively exploring the potential of combination therapies to synergize the power of ICIs with other agents [46–48]. This includes VEGF or TKI such as lenvatinib and cabozantinib [49, 50]. Promising early results from the IMbrave150, COSMIC-312, and LEAP-002 trials suggest that these combinations could further enhance patient outcomes [12, 51, 52]. The addition of TIGIT inhibitors to established regimens represents an exciting new frontier [53]. While early data from the MORPHEUS trial hint at potential benefits, larger, rigorously designed trials are necessary to definitively confirm their efficacy in this context [54]. Similarly, IMMUNIB trial, despite demonstrating promising survival benefits, warrants further investigation due to its similarities with LEAP-002 trial [55]. Undeterred by these challenges, the pursuit of even more efficacious therapies for advanced HCC remains relentless. Ongoing research strives to refine treatment options, optimize combination strategies, and ultimately improve the prognosis for patients battling this aggressive malignancy. With the continuous influx of innovative approaches and a commitment to personalized medicine, the future of first-line treatment for advanced HCC appears increasingly promising.

Sorafenib is a multitargeted tyrosine kinase inhibitor that influences angiogenesis, apoptosis, and proliferation in cancer [56, 57]. Despite promising outcomes of clinical trials, sorafenib improved OS with a minor increase of less than one year [14, 15] Moreover, sorafenib elicits a spectrum of adverse events, encapsulating diverse manifestations, and the emergence of drug resistance is incited by the substantial heterogeneity intrinsic to HCC, thereby engendering variances in therapeutic responsiveness across distinct patient cohorts [58, 59]. Due to complex and sequential events characterizing the phases of development and progression of HCC, concurrent administration of multiple therapeutic agents targeting pivotal pathways or essential molecules implicated in hepatocarcinogenesis appears to be a promising approach.

In the pursuit of novel approaches for the treatment of liver cancer, recent investigations have highlighted the immense potential for combining natural compounds with conventional anticancer treatments [60]. As a powerful compound for antitumor activities, Berberine induces G1 phase cell cycle arrest in HCC cell lines, including Huh-7 and HepG2, through inhibition of the AKT pathway leading to decreased levels of S-phase kinase-associated protein 2 (Skp2) and increased nuclear translocation of FoxO3a [61]. Apigenin derivatives have demonstrated promising anticancer properties through in silico analysis, showing their ability to inhibit critical cancer-associated targets, such as DNA polymerase theta which supports the rationale for incorporating natural small molecules into established therapies like sorafenib, to enhance treatment efficacy and overcome resistance mechanisms [62]. Additionally, these findings advocate for the broader application of similar methodologies in liver cancer, particularly in augmenting the antitumor efficacy of NK cell-based immunotherapies in conjunction with sorafenib. The combination of sorafenib and NK cell immunotherapy with natural small compounds offers a multidimensional approach to overcoming medication resistance. Natural substances can sensitize cancer cells to sorafenib, thereby increasing their susceptibility to its effects, while also enhancing the immune response through NK cell activation. This combination therapy strategy has the potential to dismantle the defenses of resistant HCC cells, resulting in more effective and comprehensive cancer management. Consequently, natural small molecules show promise as adjuvant agents in overcoming chemotherapeutic drug resistance in HCC, potentially improving the overall outcomes of treatment regimens that include sorafenib and NK cell immunotherapy.

NK cells have been empirically observed to demonstrate heightened presence within the hepatic microenvironment, undertaking pivotal roles in immune surveillance during HCC [63, 64]. Of particular significance, ligands that engage numerous activating NK cell receptors exhibit heightened levels of expression in HCC cells [65]. The disruptions in both the frequency and functional attributes of NK cells during HCC progression were emphasized in preclinical studies [55, 66]. Nevertheless, the precise mechanism by which sorafenib modulates NK cell function remains a subject of ongoing investigation and debate. In contrast, expanded and activated NK cells have demonstrated potent cytotoxicity against HCC cells, significantly enhancing the anti-tumor efficacy of sorafenib and exhibiting sustained activity even in the presence of the drug. However, accumulating evidence suggests a complex interplay between sorafenib, macrophages, and NK cells. Previous research has demonstrated that sorafenib can induce a shift in tumor-associated macrophage (TAM) phenotype towards a pro-inflammatory state, thereby promoting NK cell activation in a cytokine- and NF-κB-dependent manner [29]. Zhuang et al. demonstrated that NK cells supplemented with IL-12/15/18 significantly reduced spontaneous HCC development (*p* < 0.01) [67]. Importantly, NK cells isolated from HCC patients displayed comparable cytotoxic activity against HCC cell lines when compared to healthy controls. These findings suggest the potential application of cytokine-activated NK cells as an immunotherapeutic strategy for HCC. Therefore, improving the cytotoxicity of NK cells emerges as a notable strategy involving the administration of sorafenib in combination.

A recent study found that sorafenib has an immunomodulatory effect that enhances the ability of NK cells to kill cancer cells, such as HCC cells, by reducing MHC class-I molecule expression on HCC cells, which may facilitate sensitivity to cytotoxic responses mediated by NK cells [68]. Sorafenib may improve NK cell infiltration and activity at the tumor site by altering the dynamics of the tumor microenvironment. This collaborative approach could lead to a miscellaneous attack on tumors, with sorafenib sensitizing tumor cells to NK cell-mediated killing and NK cells benefiting from an improved tumor microenvironment for their effector functions. However, comprehensive exploration of the synergistic interplay between sorafenib and NK cells remains limited. Recently, Hosseinzadeh et al. found that sorafenib and NK cell monotherapies could not facilitate the HCC xenograft growth rate, which may be associated with the cytotoxicity of the combination therapy and the sorafenib-associated immunosuppressive burden on immunodeficient mice [69]. In contrast, we followed an engineering strategy to improve the cytotoxicity and viability of rat NK cells through the activation of the cytokines IL-12 and IL-18 before IHA administration in combination with sorafenib to boost therapeutic response. The experimental results demonstrated a significant reduction in tumor growth in the combination therapy group compared to the sorafenib monotherapy, *p*NK cell immunotherapy, and control groups, despite the absence of a synergistic effect between sorafenib and *p*NK cell immunotherapy. Furthermore, histopathological findings corroborated MRI measurements, revealing underlying changes in the tumor microenvironment, including reduced tumor viability and an increased presence of NK cells. Additionally, we investigated the association between MRI-derived quantitative features and histopathological markers in assessing the tumor microenvironment. MRI metrics demonstrated the potential to differentiate treatment effects, with entropy and skewness showing promise as predictors of tumor necrosis. While T1w MRI-derived metrics correlated well with both tumor necrosis and NK cell migration, T2w metrics exhibited weaker predictive power. These findings suggest that MRI-based quantitative analysis could serve as a non-invasive tool for monitoring treatment response and understanding tumor heterogeneity. Our findings regarding the immunomodulatory effects of sorafenib on NK cell function align with previous research. Several studies have demonstrated the ability of sorafenib to enhance NK cell cytotoxicity against HCC cells by downregulating MHC class I expression [26]. Furthermore, our observation that sorafenib can modify the tumor microenvironment to favor NK cell infiltration and activity is consistent with the emerging understanding of the drug’s immunomodulatory properties. However, our results diverge from those of Hosseinzadeh et al., who reported a lack of synergy between sorafenib and NK cells in an HCC xenograft model [69]. This discrepancy may be attributed to differences in experimental design, including the use of engineered NK cells in our study. Our findings suggest that enhancing NK cell function through activation and expansion can overcome the immunosuppressive effects observed in previous studies. The correlation between MRI-derived quantitative features and histopathological markers is a novel contribution to the field. While the use of MRI for assessing tumor response is well-established, our study provides additional insights into the potential of quantitative MRI analysis for characterizing the tumor microenvironment and predicting treatment response. These results warrant further investigation to establish their clinical utility.

Several limitations were inherent in the present investigation. First, we performed a preclinical study to investigate the therapeutic response with a single rodent HCC model that may not replicate the heterogeneity of human HCC tumors. However, the rat HCC model utilized in our study was widely adopted for oncological experiments to analyze therapeutic responses in HCC tumors and replicate the severity of the human model. A larger preclinical study integrating multiple tumor models may be beneficial to further evaluate the therapeutic response. Second, the sample size of the groups was smaller due to the nature of the preclinical study; however, histopathological analysis results supported the MRI findings and highlighted the strong support for performing phase I clinical trials. Last, we evaluated structural changes using T1w and T2w MRI data, in which the immunotherapeutic response may take a longer time to trigger stronger effects. Nevertheless, the slowing of tumor growth became visible in the following weeks of the study.

## Conclusions

Our study investigated the therapeutic response of sorafenib combined with NK cell immunochemotherapy against HCC through a preclinical model. We compared the potential therapeutic effects of this combination therapy to those of monotherapies by monitoring tumor growth non-invasively via MRI and validating the results with histopathological analysis. Our findings suggest that the combination of sorafenib and *p*NK cell immunotherapy effectively elicits an antitumor response and slows HCC progression. This approach shows considerable promise for further comprehensive studies and holds potential for translation into clinical trials to assess treatment efficacy.

## Data Availability

The datasets used and/or analyzed during the current study are available from the corresponding author on reasonable request.

## Abbreviations

FA: Flip angle
FOV: Field of view
HCC: Hepatocellular carcinoma HCC
NK: Natural killer
pNK: Cytokine activated memory-like NK cells
NSA: Number of signals acquisitions
ST: Slice thickness
TE: Echo time
TR: Repetition time
